# Declining mortality after open pelvis fracture in North America

**DOI:** 10.1007/s00590-025-04559-z

**Published:** 2025-10-13

**Authors:** Soroush Shabani, Annie Zhang, Julian Wier, Joseph T. Patterson

**Affiliations:** Keck School of Medicine of the University of Southern California, Los Angeles, USA

**Keywords:** Open pelvic fracture, Mortality, Angioembolization, Preperitoneal packing, Exploratory laparotomy

## Abstract

**Purpose:**

Open pelvis fractures are associated with a high rate of mortality and require a multidisciplinary approach to resuscitation, hemorrhage control, and fracture stabilization. The patients presenting with these injuries, practice guidelines, and use early of interventions including angioembolization (AE), exploratory laparotomy (EL), and preperitoneal pelvic packing (PPP) have changed over time. It is not known if these changes are associated with mortality.

**Methods:**

Adults presenting with an open pelvis fracture between 2017 and 2022 were retrospectively identified from the American College of Surgeons Trauma Quality Improvement Program. The primary outcome was in-hospital mortality identified by “deceased” or “expired” emergency department or hospital discharge disposition. Patient-level adjusted risk of mortality was calculated by multivariable logistic regression considering patient comorbid conditions, injury characteristics, interventions, and facility characteristics across observed years. Adjusted mortality risk relative to AE, EL, and PPP interventions was assessed per year by Chi-square and Kruskal–Wallis tests.

**Results:**

Of 10,172 eligible patients identified, 81.44% were male. The mean adjusted mortality was 10.78% and significantly decreased by 0.43% per year (*p* = 0.001). AE was performed for 4.42% of patients and did not significantly change per year. EL was performed for 8.03% of patients and decreased by 0.60%/year (*p* < 0.001). PPP was performed for 7.56% of patients and increased by 0.76%/year (*p* < 0.001). Trauma centers of mid-tier size (400–600 beds) reported increasing admissions of open pelvis fractures.

**Conclusion:**

In-hospital mortality after open pelvis fracture declined 2017–2022 as practice patterns evolved for the acute management of associated hypotension, including increased use of PPP and decreased use of EL.

**Level of evidence:**

Prognostic Level III.

**Supplementary Information:**

The online version contains supplementary material available at 10.1007/s00590-025-04559-z.

## Introduction

Open fractures of the pelvis are rare injuries associated with high rates of mortality, fracture-related infection, and long-term disability [1]. Although open fractures account for only 2–4% of pelvis fractures, patients who sustain these injuries typically present after high-energy polytrauma in hemorrhagic shock with multiorgan dysfunction [1,2]. In-hospital mortality after open pelvis fracture has been reported from 4 to 45% [1,2]. Emergent management requires a multidisciplinary approach including resuscitation, hemorrhage control, fracture stabilization, and treatment of associated injuries. Patients who survive an open pelvis fracture often require multiple surgical interventions, extensive critical care resources, long hospital stays, and intensive rehabilitation, placing a substantial burden on healthcare systems.

Care approaches to the management of patients with open fractures of the pelvis continue to evolve and to vary across centers [1,3]. Early interventions for clinically unstable patients with these injuries focus on hemorrhage control. These interventions include the application of circumferential pelvic compression devices during prehospital transport [4] or upon arrival to a trauma center [5], vascular interventions including angioembolization (AE) and resuscitative endovascular balloon occlusion of the aorta (REBOA), and emergent surgical interventions including various combinations of exploratory laparotomy (EL), preperitoneal packing (PPP), external fixation, debridement of the of open fracture, and limited internal fixation. Advanced Trauma Life Support (ATLS) guidelines published by the American College of Surgeons Committee on Trauma offer little guidance on the appropriate set, sequence, or relative efficacy of these interventions to minimize mortality after open pelvis fractures [6].

Practice guidelines for pelvis fractures published by surgical societies have changed over the past decade, as has the reported use of these interventions for unstable patients with open pelvis fracture [7–10]. It remains unknown if temporal changes in the management of open pelvis fractures have occurred among North American trauma centers or if these changes were associated with in-hospital mortality. This study sought to determine whether in-hospital mortality for patients with open fractures of the pelvis changed concurrent with shifts in the acute management of these injuries while adjusting for changes in patient characteristics, facility characteristics, and injury severity over time.

## Methods

### Data source and study population

Adults presenting with an open pelvis fracture between 2017 and 2022 were identified from retrospective review of the American College of Surgeons Trauma Quality Improvement Program (ACS-TQIP) database [11]. The ACS-TQIP contains validated data prospectively collected from over 800 participating hospitals. Abbreviated Injury Scale (AIS) diagnosis codes for pelvis fracture used to identify patients with open pelvis fractures and stratify injury severity according to the Tile classification as type A (stable), type B (incomplete posterior pelvic ring disruption), or type C (complete posterior pelvic ring disruption; Supplemental Table 1) [12]. Concomitant injury characteristics associated with mortality after pelvis fracture were categorized by AIS codes including bowel injury requiring surgery, bladder injury requiring surgery, and severe head injury (defined as AIS in head region >  = 4). Injury Severity Score (ISS) was recorded. Acute treatments for hemodynamic instability including AE, EL, and PPP were identified by International Classification of Diseases, Tenth Revision, Clinical Modification (ICD-10-CM) procedural codes. The burden of comorbid medical conditions was calculated using the Charlson Comorbidity Index (CCI) calculated with ICD-10 codes [13]. Treating facility characteristics of interfacility transfer, bed size, hospital type, and primary method of payment were collected. TQIP years prior to 2017 were excluded due to concerns that changes in variable definitions and classification of procedures and diagnoses secondary to the transition to ICD-10-CM would introduce confounding. This investigation was classified as exempt by the institutional review board as all patient information was de-identified in accordance with the Health Insurance Portability and Accountability Act.

**Table 1:** Trends in open pelvic fracture injury severity, interventions, and subsequent mortality

Year	2017 N = 1342	2018 N = 1395	2019 N = 1439	2020 N = 1905	2021 N = 2086	2022 N = 2005	All years N = 10,172	Average percent change	*p* value
Age (years)	35.22 ± 16.29	35.29 ± 16.22	33.95 ± 15.53	32.54 ± 14.12	32.88 ± 14.59	33.76 ± 14.36	33.78 ± 15.09	− 0.81	** < 0.001**
Sex									**0.003**
Male	1082 (80.63%)	1123 (80.5%)	1124 (78.11%)	1579 (82.89%)	1716 (82.26%)	1660 (82.79%)	8284 (81.44%)	0.433	
Female	260 (19.37%)	272 (19.5%)	315 (21.89%)	326 (17.11%)	370 (17.74%)	343 (17.11%)	1886 (18.54%)	− 0.453	
Nonbinary						2 (0.1%)	2 (0.1%)	0.02	
CCI	0.48 ± 1.09	0.45 ± 1.06	0.38 ± 0.99	0.28 ± 0.8	0.31 ± 0.85	0.32 ± 0.84	0.36 ± 0.93	− 6.76	** < 0.001**
*Injury characteristics*									
Tile classification, Open pelvic fracture									** < 0.001**
A	942 (70.19%)	930 (66.67%)	1035 (71.92%)	1416 (74.33%)	1623 (77.8%)	1493 (74.46%)	7439 (73.13%)	0.854	
B	282 (21.01%)	306 (21.94%)	260 (18.07%)	320 (16.8%)	274 (13.14%)	307 (15.31%)	1749 (17.19%)	− 1.14	
C	118 (8.79%)	159 (11.4%)	144 (10.01%)	169 (8.87%)	189 (9.06%)	205 (10.22%)	984 (9.67%)	0.286	
Severe head injury (AIS > = 4)	68 (5.07%)	77 (5.52%)	88 (6.12%)	91 (4.78%)	97 (4.65%)	97 (4.84%)	518 (5.09%)	− 0.046	0.398
Bowel injury	319 (23.77%)	328 (23.51%)	377 (26.2%)	551 (28.92%)	641 (30.73%)	615 (30.67%)	2831 (27.83%)	1.38	** < 0.001**
Bladder injury	71 (5.29%)	69 (4.95%)	82 (5.7%)	111 (5.83%)	101 (4.84%)	126 (6.28%)	560 (5.51%)	0.199	0.353
ISS	22.87 ± 12.68	23.67 ± 13.83	23.52 ± 13.3	22.64 ± 12.72	22.68 ± 12.67	23.25 ± 12.82	23.06 ± 12.97	0.37	0.309
Facility characteristics									
Interfacility transfer	208 (15.5%)	217 (15.56%)	231 (16.05%)	312 (16.38%)	324 (15.53%)	310 (15.46%)	1602 (15.75%)	− 0.008	0.965
Payment									** < 0.001**
Medicaid	396 (29.51%)	436 (31.25%)	468 (32.52%)	681 (35.75%)	822 (39.41%)	775 (38.65%)	3578 (35.17%)	1.83	
Not billed	14 (1.04%)	4 (0.29%)	4 (0.28%)	12 (0.63%)	8 (0.38%)	7 (0.35%)	49 (0.48%)	− 0.139	
Self-pay	280 (20.86%)	307 (22.01%)	333 (23.14%)	464 (24.36%)	407 (19.51%)	390 (19.45%)	2181 (21.44%)	− 0.283	
Private insurance	462 (34.43%)	482 (34.55%)	468 (32.52%)	551 (28.92%)	625 (29.96%)	629 (31.37%)	3217 (31.63%)	− 0.611	
Medicare	90 (6.71%)	84 (6.02%)	83 (5.77%)	74 (3.88%)	89 (4.27%)	93 (4.64%)	513 (5.04%)	− 0.414	
Other government	54 (4.02%)	40 (2.87%)	41 (2.85%)	71 (3.73%)	78 (3.74%)	69 (3.44%)	353 (3.47%)	− 0.116	
Other	46 (3.43%)	42 (3.01%)	42 (2.92%)	52 (2.73%)	57 (2.73%)	42 (2.09%)	281 (2.76%)	− 0.267	
Hospital type									0.526
Nonprofit	1212 (90.31%)	1271 (91.11%)	1288 (89.51%)	1740 (91.34%)	1893 (90.75%)	1809 (90.22%)	9213 (90.57%)	− 0.018	
For-profit	130 (9.69%)	124 (8.89%)	151 (10.49%)	165 (8.66%)	193 (9.25%)	196 (9.78%)	959 (9.43%)	0.018	
Bed number									** < 0.001**
< 200	94 (7%)	53 (3.8%)	65 (4.52%)	87 (4.57%)	123 (5.9%)	127 (6.33%)	549 (5.40%)	− 0.134	
201–400	304 (22.65%)	319 (22.87%)	341 (23.7%)	410 (21.52%)	468 (22.44%)	412 (20.55%)	2254 (22.16%)	0.421	
401–600	382 (28.46%)	428 (30.68%)	450 (31.27%)	573 (30.08%)	680 (32.6%)	666 (33.22%)	3179 (31.25%)	0.95	
> 600	562 (41.88%)	595 (42.65%)	583 (40.51%)	835 (43.83%)	815 (39.07%)	800 (39.9%)	4190 (41.19%)	− 0.396	
Interventions									
AE	63 (4.69%)	61 (4.37%)	63 (4.38%)	97 (5.09%)	92 (4.41%)	74 (3.69%)	450 (4.42%)	− 0.201	0.44
PPP	77 (5.74%)	88 (6.31%)	91 (6.32%)	145 (7.61%)	177 (8.49%)	191 (9.53%)	769 (7.56%)	0.758	** < 0.001**
EL	138 (10.28%)	114 (8.17%)	132 (9.17%)	143 (7.51%)	144 (6.9%)	146 (7.28%)	817 (8.03%)	− 0.600	**0.003**
Outcomes									
Preadjusted mortality	142 (10.58%)	170 (12.19%)	161 (11.19%)	203 (10.66%)	204 (9.78%)	217 (10.82%)	1097 (10.78%)	0.93	0.372
Adjusted mortality (%)*	10.88 ± 0.46	12.04 ± 0.50	11.48 ± 0.46	10.31 ± 0.38	10.10 ± 0.35	10.51 ± 0.36	10.78 ± 0.08	− 0.43	**0.001**

CCI, Charlson Comorbidity Index; severe head injury = defined as AIS head >  = 4; ISS = Injury Severity Score; AE = angioembolization; PPP = preperitoneal packing; EL = exploratory laparotomy, *reported with standard error

### Study endpoints

The primary outcome was mortality identified by “deceased” or “expired” emergency department or hospital discharge disposition.

### Statistical analysis

Available patient demographics, hospital factors, and rates of medical comorbidities were compared between years. To minimize confounding due to differences in patient comorbid conditions, injury characteristics, interventions, and facility characteristics between years, the patient-level adjusted risk of in-hospital mortality was calculated across all years by multivariable logistic regression considering sex, age, severe head injury, Tile classification, bowel injury requiring surgery, bladder injury requiring surgery, ISS, CCI, interfacility transfer, hospital bed number, hospital type, and primary method of payment as covariates. The adjusted mortality as well as absolute incidence of AE, EL, and PPP was then compared across study years. The mean year to year percent change was also reported. A Chi-square test was used to analyze categorical variables. The Kruskal–Wallis test was used for continuous variables. A *p* value < 0.05 was considered significant. Missing values were not imputed. Statistical analyses were performed with R (version 4.4.1, R foundation, Vienna, Austria).

## Results

Of the 10,172 eligible patients identified, 81.44% of were male. The portion of patients with open pelvis fractures who were male significantly increased by 0.43%/year (*p* = 0.003). The average age was 33.78 ± 15.09 years and significantly decreased by 0.81%/year (*p* < 0.001). The average CCI was 0.36 ± 0.93 and similarly decreased by 6.76%/year (*p* < 0.001) (Table 1). The mean ISS was 23.06 ± 12.97 and did not significantly change over the study period (Table 1). Tile A stable pelvis fractures represented the most common fracture pattern (73.13%) and increased in relative frequency by 0.85%/year (*p* < 0.001). Bowel injury requiring surgery was identified in 27.83% of patients and significantly increased over time by 1.38%/year (*p* < 0.001). The prevalence of bladder injury and severe head injury did not significantly vary over the study period. At the start of the observed period in 2017, private insurance was the most common form of payment (34.43%). However, Medicaid surpassed private insurance as the most common payment method in 2020, accounting for 38.65% of cases in 2022 and increasing by 1.83%/year (*p* < 0.001).

The characteristics of facilities caring for open pelvis fractures also changed over the observed. Mid-tier sized hospitals (400–600 beds) experienced growth in the admission of adults with open pelvis fractures by 0.95%/year (*p* < 0.001) while large hospitals (> 600 beds) experienced a 0.39% decline in the admission of patients with these injuries. The for-profit status of hospitals admitting open pelvis fractures did not significantly change over time (mean 90.57%, + 0.02%/year, *p* = 0.526). The incidence of interfacility transfer status did not significantly change over time (mean 15.75%, − 0.008%/year, *p* = 0.965).

**Fig. 1:**
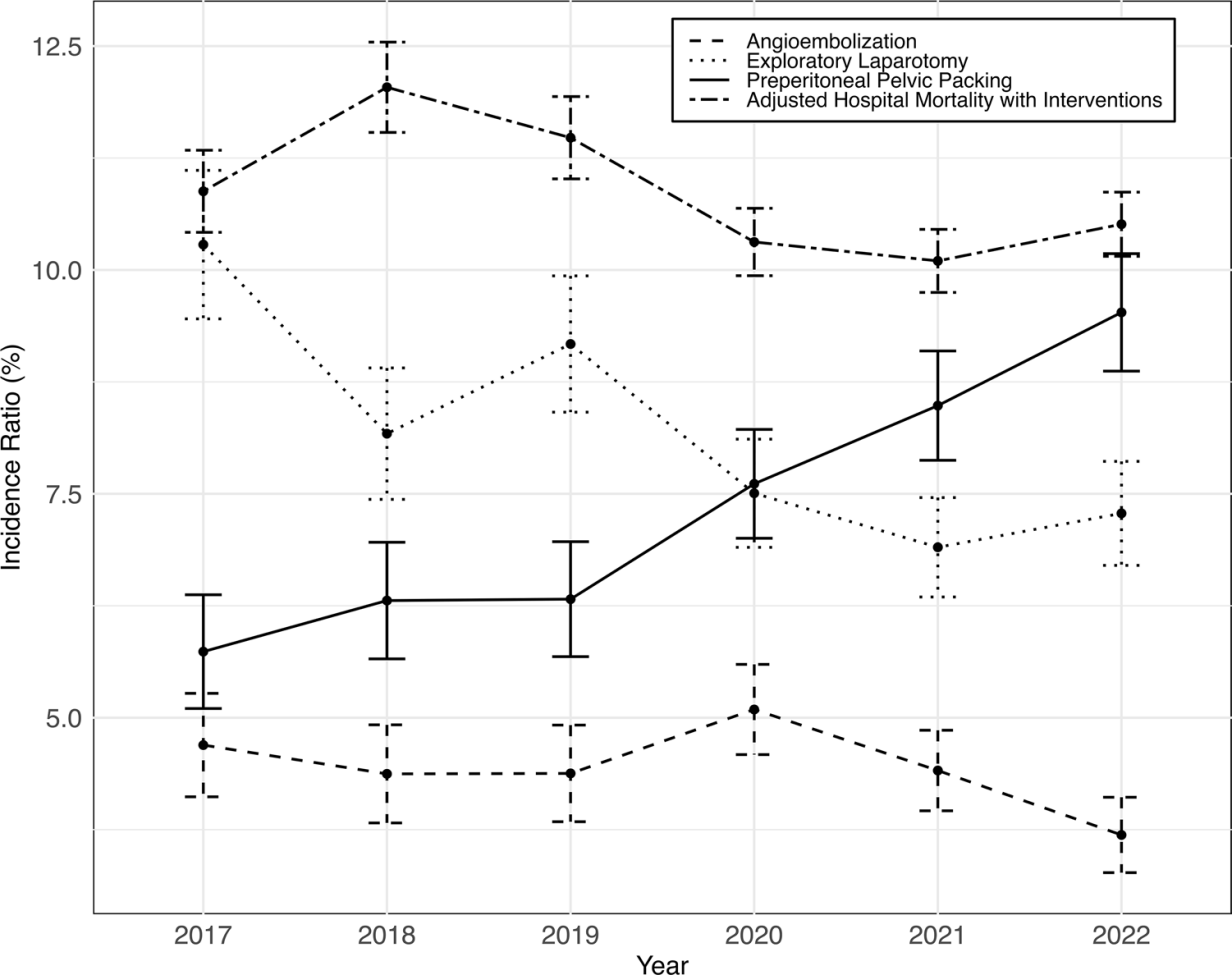
Incidence ratio with standard error of interventions per year

The unadjusted rate of mortality after open pelvis fracture was 10.78% and did not significantly change over the observed period (0.93%/year, *p* = 0.372). The mean adjusted mortality was 10.78% and significantly decreased by 0.43%/year (*p* = 0.001) (Fig. 1). Temporal changes in the use of acute interventions to manage hemodynamic instability associated with an open pelvis fracture were also observed. The incidence of AE was 4.42% and did not significantly change across years. The incidence of preperitoneal pelvic packing was 7.56% and increased by 0.76%/year (*p* < 0.001). The incidence of EL was 8.03% and decreased 0.60%/year (*p* < 0.001).

## Discussion

The characteristics of patients with open pelvis fractures presenting to North American trauma centers changed 2017–2022, as did the severity of their injuries, the facilities providing their care, the treatments they received, and their risk of mortality during the index hospital admission. Over the observed period, adults with open pelvis fractures became increasingly male and younger with a less severe burden of medical conditions and more frequently had public health insurance. Injury characteristics also changed, including a minor decline in unstable Tile B and C fracture patterns but an increase in serious concomitant abdominal injuries requiring surgical management. Mid-tier sized hospitals saw the greatest increase in the volume of open pelvis fractures despite no significant change in interfacility transfer patterns. The incidence of in-hospital mortality after open pelvis fracture declined 0.43% per year even after adjusting for the changes in patient, injury, and facility characteristics over time. This improvement in adjusted in-hospital mortality occurred concurrently with increased use of preperitoneal packing and decreased use of exploratory laparotomy in the acute resuscitation period.

Open pelvis fractures are often associated with multisystem trauma: Pelvis fractures occur in nearly 25% of polytrauma victims and up to 75% of pelvis fracture patients are polytraumatized, factors which complicate the standardization of treatment algorithms for pelvis fracture [3,14–16]. Major hemorrhage and multiple injuries remain the main predictors of mortality in patients with pelvis fractures [17]. Resuscitation and hemorrhage control also continue to be critical tenants of early care as recommended by ATLS guidance [6]. Timely interventions including circumferential pelvic compression, AE, and PPP (with the identification and treatment of associated injuries and fracture stabilization) have been reported with improved survival rates in multicenter cohort studies [18]. This positive trend continues a trajectory of decreasing mortality since the 1970s, when uncontrolled hemorrhage or late sepsis from fracture-related infection was associated with mortality up to 50% [1]. The establishment of coordinated trauma systems, early aggressive fracture management, and selective fecal diversion occurred as mortality from open pelvis fractures declined to 18% in the 1990s. [19] More recent estimates of mortality after open pelvis fractures vary widely from 4 to 50% [3,19], making it difficult to determine if progress in the quality of care continues. The present study identified an adjusted mortality of 10.8%, decreasing by 0.43% per year after 2017, which may reflect steady progress in the quality of trauma care for open pelvis fractures.

While AE and PPP are effective tools for initial hemorrhagic control following pelvis fracture, the role for EL in the context of an isolated open pelvis fracture may be less clear. PPP may offer shorter time to intervention than AE in centers where in-house personnel are more readily available to perform the pelvic packing than angiography consultants on home call. However, AE is often still needed after PPP to achieve hemodynamic stability in critical patients [20–23]. A systematic analysis by McDonogh et al. identified a similar mortality rate between AE and PPP [20], while registry studies have suggested higher rates of mortality, venous thromboembolism, and complications of subsequent surgical procedures following PPP compared to AE [24,25]. As an alternative intervention directed at hemorrhage control and resuscitation, EL may be associated with a greater risk of hemorrhage-associated mortality in major pelvic trauma. Verbeek et al. reported hemorrhage-associated mortality in 29% of patients with open pelvic fractures after EL across 11 centers in a retrospective study [26]. However, these laparotomy patients had higher ISS and lower blood pressure compared to the angiography comparison group, which may reflect confounding by indication for emergent diagnosis and management of chest and/or abdominal sources of hemorrhage causing hemodynamic instability. Katsura et al. explored this through adjustment for comorbidities, ISS, AIS of all body regions, and systolic blood pressure, reporting no difference in mortality rate between hemodynamically unstable patients with a pelvis fracture receiving initial therapeutic intervention by AE versus EL [27]. EL is a traditional primary intervention for hemorrhage control, but in open pelvic fracture is associated with high morbidity and may often be nontherapeutic [28–30].

While published guidelines address the use of AE, EL, and PPP for hemorrhage control in the context of pelvis fracture, recommendations for initial management differ between guidelines. The 2011 Eastern Association for the Surgery of Trauma (EAST) endorsed PPP as an effective adjunct to the gold standard of AE [31]. The 2016 Western Trauma Association recommended external pelvic stabilization with a binder, followed by AE and/or PPP for hemodynamically unstable pelvis fractures [9]. Similarly, the 2016 National Institute for Healthcare Excellence and 2018 British Orthopaedic Association guidelines favored AE as first-line therapy in patients needing EL, with PPP used intraoperatively if EL is performed [32]. In contrast, the World Society of Emergency Surgery advocated PPP with EL as initial management, reserving AE for ongoing hemorrhage [33]. The 2019 ATLS update supports EL with intraperitoneal packing, and PPP or AE for unstable patients, noting lower mortality with PPP-initial approaches [6]. Both the American Society for Surgery of Trauma and the pan-European Task Force highlight PPP as a bridge to AE or a standalone option when AE is unachievable, also recommending REBOA for noncompressible life-threatening bleeding [34,35]. Despite differences in initial management, these guidelines underscore a shared consensus on the utility and importance of both AE and PPP in achieving hemorrhage control and reducing mortality.

Treatment strategies, multidisciplinary coordination, emergency medical systems, and other aspects of open pelvis care have evolved in recent years. The reported rates of PPP and AE interventions for pelvis fractures vary widely, as do clinical protocols for pelvic trauma between institutions [26]. Historically, AE had been more common in the USA and PPP utilized more often in Europe [20,36,37]. The rate of AE has also increased in the USA, with an average incidence of 3.3% between 2008 and 2010 increased to 11% between 2010 and 2016 [36,38]. Pelvic packing interventions were eventually integrated into trauma protocols more commonly, coinciding with a large nonrandomized study demonstrating improved outcomes and decreased time to intervention with PPP [26]. Fitzgerald et al. reported an incidence of 13.3% for patients with pelvic packing from 2002 to 2012 in a single-center review [39]. Early guidelines for unstable pelvis fractures also supported immediate laparotomy as the initial therapeutic intervention [28]. However, recent studies have found that initial embolization may reduce the rate of nontherapeutic EL [40,41]. This study identified a rise in PPP by 1.6% per year and AE by 0.5% per year. In contrast, EL has decreased in utilization by 0.54% per year. Along with these changes, the adjusted mortality rate has decreased by 0.19%/year.

Mid-tier size hospitals have reported increasing admissions for open pelvis fractures, which occurred concurrently with no significant change in the rate of interfacility transfers. Studies suggest that access to advanced interventions and personnel may be more important than size alone. For instance, large hospitals (> 600 beds) have shown higher rates of AE, likely due to in-house or rapidly available angiography services [36]. Similarly, improved outcomes for major trauma patients have been observed in high-volume hospitals, though not across all trauma populations [42,43]. Concurrently, the prevalence of Medicaid insurance increased while private and self-pay cases declined, a trend consistent with Medicaid expansion after the Affordable Care Act and reported in other orthopedic trauma literature [44,45]. Importantly, uninsured pelvis fracture patients experienced significant disparities in diagnostic imaging and procedures, along with more than double the mortality rate compared to insured patients (11.6% vs. 5.0%, respectively) [46]. These difference were most pronounced for invasive and resource-intensive procedures, such as computed tomography (CT) of the abdomen and AE, which are pertinent for open pelvis fracture diagnosis and management [46]. Together, these findings suggest that while hospital size alone may not drive outcomes, access to advanced care and insurance coverage are critical for timely, appropriate management of open pelvis fractures.

The present investigation observed the COVID-19 pandemic and its impact on trauma admissions and management patterns. While the overall incidence of pelvis fractures remained stable, nonsignificant declines in the number of open pelvis fractures occurred during the pandemic lockdown periods [47]. The first decline in open pelvis fracture cases occurred between 2020 and 2021, despite an increase of 16 hospitals and over 75,000 contributing to the TQIP database during that period [48]. This shift was marked by a decrease in admissions to nonprofit hospitals and a rise in for-profit hospital admissions, reflecting a broader trend during COVID-19 of acute trauma cases managed at mid-sized hospitals while admissions to large hospitals declined [49]. Interfacility transfers for pelvis fracture also declined between 2020 and 2021 [49]. Consequently, there was a drop in procedures such as AE and EL, which often require greater resources typically available at larger hospitals. In contrast, PPP use increased, likely due not only to its expanding role in acute trauma care but also to its ease of implementation and availability in smaller facilities with less specialized personnel in eras of decreased interfacility transfer capacity and volume.^33,43^

This study has limitations including those inherent to retrospective designs and previously abstracted clinical data. The use of AIS and ICD coding to stratify and compare patients subjects the inferences to classification bias. The sample was truncated due to the adoption of ICD-10-CM in October 2015, and though robust data exist in TQIP prior to this, a clear impact of classification bias was observed between ICD-9 and ICD-10. The inability to review radiograph and CT imaging to characterize fracture patterns limits internal validity. The potential for alternate forms of selection bias, inaccurately coded values, and representation bias in the TQIP database must be acknowledged. Nonetheless, this study is strengthened by an outcome that is not censored, prospective and systematically abstracted data using defined protocols and quality control methods, and the high representation of trauma centers providing care for these injuries in the studied region. The robust, prospective data source and association observed warrant prospective investigation of alternate protocols for pelvis fracture management.

In conclusion, in-hospital mortality after open pelvis fracture declined from 2017 to 2022 as practice patterns evolved for the acute management of associated hypotension, including increased use of PPP and decreased use of EL.

## Supplementary Information

Below is the link to the electronic supplementary material.

Supplementary file1 (DOCX 15 KB)

## Data Availability

Data was extracted from the TQIP database.
