# Supplementary material for: Declining mortality after open pelvis fracture in North America
Source: Eur J Orthop Surg Traumatol. 2025 Oct 13;35(1):432. doi: 10.1007/s00590-025-04559-z (PMC12518424; doi:10.1007/s00590-025-04559-z)
Supplement: Supplementary file 1 — Supplementary file1 (DOCX 15 KB) [file 590_2025_4559_MOESM1_ESM.docx]

| Supplemental Table 1: Diagnostic and procedural codes used to query patients | |
| --- | --- |
| Term | Code |
| Abbreviated Injury Scale (AIS) | |
| Tile A, Open Fracture | 856152 |
| Tile B, Open Fracture | 856162 |
| Tile C, Open Fracture | 856174 |
| Bladder Injury Requiring Operation | 540624, 540625, 540626, 540640 |
| Bowel Injury Requiring Operation | 540820, 540822, 540823, 540824, 540826, 541020, 541021, 541022, 541023, 541024, 541025, 541026, 541028, 541420, 541422, 541423, 541424, 541426, 541499 |
| International Classification of Diseases, 10th Revision (ICD-10) | |
| Angioembolization | 04LC3DZ, 04LC4DZ, 04LD3DZ, 04LD4DZ, 04LE3DZ, 04LE4DZ, 04LF3DZ, 04LF4DZ, 04LH3DZ, 04LH4DZ, 04LJ3DZ, 04LJ4DZ |
| Preperitoneal Pelvic Packing | 2W43X5Z, 2W13X6Z |
| Exploratory Laparotomy | 0DJW0ZZ, 0DJ00ZZ, 0DJ60ZZ, 0DJD0ZZ, 0DJU0ZZ, 0WJG0ZZ, 0WJJ0ZZ, 0WJP0ZZ, 0WJR0ZZ |
